# Key enzymes and proteins of crop insects as candidate for RNAi based gene silencing

**DOI:** 10.3389/fphys.2015.00119

**Published:** 2015-04-22

**Authors:** Vijaya Sudhakara Rao Kola, P. Renuka, Maganti Sheshu Madhav, Satendra K. Mangrauthia

**Affiliations:** Department of Biotechnology, Directorate of Rice Research, ICAR-Indian Institute of Rice ResearchHyderabad, India

**Keywords:** siRNA, off target, insecticide, resistance, dicer

## Abstract

RNA interference (RNAi) is a mechanism of homology dependent gene silencing present in plants and animals. It operates through 21–24 nucleotides small RNAs which are processed through a set of core enzymatic machinery that involves Dicer and Argonaute proteins. In recent past, the technology has been well appreciated toward the control of plant pathogens and insects through suppression of key genes/proteins of infecting organisms. The genes encoding key enzymes/proteins with the great potential for developing an effective insect control by RNAi approach are actylcholinesterase, cytochrome P450 enzymes, amino peptidase N, allatostatin, allatotropin, tryptophan oxygenase, arginine kinase, vacuolar ATPase, chitin synthase, glutathione-S-transferase, catalase, trehalose phosphate synthase, vitellogenin, hydroxy-3-methylglutaryl coenzyme A reductase, and hormone receptor genes. Through various studies, it is demonstrated that RNAi is a reliable molecular tool which offers great promises in meeting the challenges imposed by crop insects with careful selection of key enzymes/proteins. Utilization of RNAi tool to target some of these key proteins of crop insects through various approaches is described here. The major challenges of RNAi based insect control such as identifying potential targets, delivery methods of silencing trigger, off target effects, and complexity of insect biology are very well illustrated. Further, required efforts to address these challenges are also discussed.

## Introduction

Insects are considered as the most significant biotic constraints affecting plants and animals life worldwide. Insects not only cause direct loss to agricultural production, but also indirectly as vectors for various plant pathogens. They belong to class Insecta or Hexapoda under the largest class of the major invertebrate phylum Arthropoda, with more than 30 orders (Gullan and Cranston, 2010; http://espacepourlavie.ca/en/chart-orders-insects).

The physiology of insects comprises several important biological systems such as digestive, circulatory, respiratory, muscular, endocrine, nervous and reproductive, which are governed by numerous proteins including enzymes. The digestive system comprises of three sections—foregut (stomodeam), midgut (mesenteron), and hindgut (proctodeam). Various enzymes are involved in the digestion process; e.g., amylase helps to break down carbohydrates, invertase breaks sucrose into monosaccharides, lipase digests lipids, pepsin, trypsin, chymotrypisin, carboxypeptidase digest proteins, and nucleases digest nucleic acids. Digestive proteinases inhibitors (especially serine PIs, cysteine PIs) have been effectively utilized for insect-control (Lehane and Billingsley, 1996). The circulatory system of insects doesn't possess well defined veins or arteries. Insect blood known as haemolymph flows freely through the body cavity and makes direct contact with organs and tissues (Chapman, 1998). It is involved in transports of hormones, nutrients, and wastes and plays important role in osmoregulation, temperature control, immunity, and skeletal function. Phenoloxidase enzyme is present throughout the body of insects including the open circulatory system of haemolymph (Reiko and Ashida, 1986). The phenoloxidase in insect haemolymph occurs as a proenzyme, prophenoloxidase which can be activated by an activator present in hemolymph and cuticle (Ashida et al., 1982). Circulatory system is known to play essential role in the molting process also (McGavin, 2001; Johnson and Triplehorn, 2005).

The respiratory system of insects resembles to other organisms as they exhale carbon dioxide, a waste product of cellular respiration. Oxygen is delivered to the cells directly through respiration, and not carried by blood (Hetz and Bradley, 2005). The basic unit of insect muscular system is the sarcomere that contains myofibrils inside the cytoplasm known as sarcoplasm. The covering membrane is the sarcolemma. Alpha-glycerophosphate dehydrogenase enzyme is important in the glycerophosphate cycle of insects flight muscle (Healy et al., 2004). The myosin heads are enzymes (ATPases) which interact with actin filaments to produce force and movement. Several key enzymes / proteins are involved in the insect reproductive system among which two enzymes namely angiotensin-converting enzyme (ACE) (Isaac et al., 1999) and juvenile hormone (JH) acid methyltransferase (JHAMT) are well studied in several insect species (Sparagana et al., 1985). Insects molting and metamorphosis are primarily under the control of two insect hormones, ecdysone and JH (Shinoda and Itoyama, 2003). Acetylcholinesterase (AChE) is an important enzyme in the central nervous system of insects. The important neurotransmitters such as acetylcholine and dopamine are released at synapses of nervous system to control the insects metabolism (Thany et al., 2010). Allatostatin and allatotropin are major insect neuropeptides which inhibit the biosynthesis of juvenile hormones (Lungchukiet et al., 2008; Abdel-latief and Hoffmann, 2014). Generally, insecticides control the insects by targeting crucial enzymes or proteins involved in different biological processes described above. Such insecticides have been classified based on their mode of action. Although, there are effective chemicals available for the control of insects, keeping in view of environmental pollution, ecological imbalance, and biohazard effects, it is necessary to find novel ways of insects control which includes RNA interference (RNAi) or RNA silencing.

RNA interference is a well described gene regulatory mechanism that controls the coding transcript level (mRNA) by either suppressing transcription (transcriptional gene silencing or TGS) or by activating a homology based mRNA degradation process (post-transcriptional gene silencing or PTGS) (Tomari and Zamore, 2005; Vaucheret, 2006; Agarwal and Mangrauthia, 2013). The process involves the production of double stranded RNAs (dsRNA) of target gene which is processed into 21–24 nt RNA duplexes by the RNase III enzyme dicer and its homologs (Zamore et al., 2000; Scott et al., 2013). These siRNAs are then incorporated into a multi-subunit endonuclease silencing complex called RNA-induced silencing complex (RISC). Argonaute proteins, the core catalytic components of RISC, use siRNA as a guide to recognize and degrade the complementary gene or mRNA (Hammond et al., 2000). Recent studies showed that transgenic plants harboring dsRNAs against insect target genes showed resistance/tolerance against insects, opening the way for a new generation of insect-resistant crops (Agarwal et al., 2012). The viable level of insect resistance (resistance of plants toward insects) can be achieved through RNAi by producing insect gene specific RNAi triggers; i.e., small interfering RNAs (siRNAs) or dsRNAs in transgenic plants (Price and Gatehouse, 2008). Among the protein based approaches, insecticidal proteins from the bacterium *Bacillus thuringiensis* (Bt) have been utilized to control some key pests through transgenic and non-transgenic methods in various crops. RNAi, an RNA based approach has great potential on crop protection against lepidopteron and coleopteran pests and likely to be taken up for applications where Bt based approaches have proven difficult, for example protection against flies (dipteran), and sap-sucking homopteran pests (Ruiz-Medrano et al., 1999; Lucas et al., 2001). In order to achieve RNAi based insect resistance, several dsRNA/siRNA delivery methods have been developed to target key proteins/enzymes of insects (Yu et al., 2013).

Despite success of this technology, there are challenges that need to be addressed to make this more effective in coming years. Major concerns are developing efficient delivery methods and testing them *in vivo* through artificial diets, identification and characterization of RNAi machinery in insects, studying the off target effects on non-target organism, and durability of resistance. Further, more information on key proteins/enzymes involved in essential biological processes of insects would be important in insect control using RNAi. In spite of these concerns, there is no doubt that researchers and farmers have reasons to look forward to a new era of insect control (Gordon and Waterhouse, 2007).

## Key enzymes of insects

The enzymes in insect's life cycle play central role in different biological processes such as digestive mechanism, defense mechanism, locomotion, feeding methods, temperature control, growth, and development etc. The list of such enzymes is given in Table 1.

**Table 1:** **Key enzymes and their role in insects life cycle**.

**S.No**	**Enzyme name**	**Function**	**Location**	**References**
**DIGESTIVE SYSTEM**				
1	Serine proteinases	Proteolytic enzymes having a serine and a hsitidine in the active site	Gut	Yan et al., 2001
2	Cysteine proteinases	Proteolytic enzymes having cysteine in the active site	Midgut	Koiwa et al., 2000
3	Aspartic proteinases	Proteolytic enzymes hydrolyzing internal peptide bonds in proteins	Small intestine (posterior midgut)	Lehane and Billingsley, 1996; Balczun et al., 2012
4	Trypsin	Proteolytic enzyme preferentially cleaves protein chains on the carboxyl side of basic amino acids	Gastric coeca and midgut	Schoofs et al., 2002
5	Chymotrypsin	Proteolytic enzyme that cleaves peptide amide bonds where the aromatic amino acids are at carboxyl side	Midgut	Lehane and Billingsley, 1996
6	Amino peptidases	Proteolytic enzymes hydrolyzing single amino acid from the N-terminus of the peptide chain	Midgutepithelium	Lehane and Billingsley, 1996; Sivakumar et al., 2007
7	Carboxypeptidases	Proteolytic enzymes hydrolyzing single amino acid from the C-terminus of the peptide chain	Whole gut	Lehane and Billingsley, 1996; Bown and Gatehouse, 2004
8	Collagenase	Endopeptidases that break the peptide bonds in collagen	Gut	Lecroise et al., 1979
9	Keratinases	Proteolytic enzymes that breakdown keratin protein	Midgut	Christeller et al., 1994
10	Elastases	Proteolytic enzymes that hydrolyse elastin protein	Midgut	Lehane and Billingsley, 1996
11	Amylases	Enzymes that catalyze the hydrolysis of starch and other polysaccharides	Midgut	Lehane and Billingsley, 1996
12	Glucosidases	Enzymes that catalyze the hydrolysis of di- and oligosaccharides	Midgut	Swingle, 1925; Lehane and Billingsley, 1996
13	Maltase	Enzyme that catalyzes the hydrolysis of the disaccharide maltose	Midgut	Swingle, 1925
14	Invertase	Enzyme that catalyzes the hydrolysis of of sucrose	Midgut	Swingle, 1925
15	Cellulase	Enzyme that catalyzes the breakdown of cellulose	Gut	Fischer et al., 2013
16	Xylanase	Enzyme that catalyzes the hydrolysis of xylose	Gut	Lehane and Billingsley, 1996; Brennan et al., 2004
17	Beta Glucanase	Enzyme that catalyzes the hydrolysis of glucan	Larval midgut	Bragatto et al., 2010
18	Lipases	Enzymes that catalyze the hydrolysis of fats (lipids)	Midgut	Swingle, 1925
**DEFENSE**				
19	Catalase	The antioxidant enzyme that converts H_2_O_2_ to water and oxygen	All tissues	Fraga et al., 2013
**METABOLISM**				
20	*Lactate dehydrogenase*	Enzyme that catalyzes the lactic acid dehydrogenation	Insect muscles	Kitto and Briggs, 1962
21	Succinate dehydrogenase	Enzyme that catalyzes the succinic acid dehydrogenation	Insect muscles	Gorbachev et al., 2013
22	NADH oxidase	Oxidative enzyme involved in oxidation/ reduction	Respiratory tissues	Shappirio, 1974
23	Succinate-cytochrome c reductase	Oxidative enzyme involved in oxidation/ reduction	Respiratory tissues	Shappirio, 1974
24	NADH-cytochrome c reductase	Oxidative enzyme involved in oxidation/ reduction	Respiratory tissues	Shappirio, 1974
25	*Pyruvate carboxylase*	Enzyme that catalyzes the ATP-dependent carboxylation of pyruvate to oxaloacetate	All insect flight muscles	Crabtree et al., 1972
26	*Hexokinase*	Enzyme that catalyzes the phosphorylation of hexose	Insect muscles	Fraga et al., 2013
27	Protein phosphatase 5 (PP5)	Enzyme that catalyzes the de-phosphorylation of proteins	Almost in all tissues	Chen et al., 2014
28	Cytochrome P450 enzymes (CYPs)	Group of enzymes involved in oxygenation, detoxification processes, and synthesis and degradation of ecdysteroids and juvenile hormones	Virtually all insect tissues	Feyereisen, 1999
29	Adenylate kinase	Phosphotransferase enzyme that catalyzes the synthesis of ATP	Virtually all insect tissues	Chen et al., 2012
30	Glycogen synthase	Enzyme that catalyzes the conversion of glucose to glycogen	Embryonic tissues	Tang et al., 2012; Fraga et al., 2013
31	*Glycogen Phosphorylase*	Enzyme that catalyzes the breakdown of glycogen	Insect muscles and fat bodies	Steele, 1982; Tang et al., 2012; Fraga et al., 2013
32	Tyramine-mono-oxygenase	Enzyme that catalyzes the synthesis of the octopamine	Nervous tissues	Hess et al., 2008
33	Juvenile hormone (JH) acid methyl-transferase	Enzyme involved in reproduction diapause, and polyphenisms	All insect tissues particularly in reproductive tissues	Shinoda and Itoyama, 2003
34	Acetylcholinesterase	Enzyme that catalyzes the hydrolysis of the acetylcholine	Nervous tissues	Yang et al., 2010
35	Chitinase	Enzyme that catalyzes the hydrolysis of chitin	Molting fluids	Arakane and Muthukrishnan, 2010

## Key regulatory and structural proteins of insects

Proteins are biological molecules imparting essential roles in insect growth and metabolism. In addition to their role as enzymes, structural, and regulatory proteins are also crucial to complete the life cycle of insects. Several insect proteins have been characterized and reported, few are listed in Table 2.

**Table 2:** **Key proteins and their function in insect life cycle**.

**S.No**	**Protein**	**Insect**	**Function**	**References**
1	Sensory neuron membrane proteins -Apo1	*Bombyx mori, Chilo suppressalis*	Involved in pheromone specific olfactory sensory mechanism	Rogers et al., 2001; Liu et al., 2013
2	Odorant binding proteins	*Loxostege sticticalis, Cydia pomonella*	Involved in transporting semiochemicals across the sensillum lymph to olfactory receptors	Yin et al., 2012; Ayme-Southgate et al., 2013
3	Larval Hemolymph Protein (LHP)	*Drosophila*	Involved in larval development	Beverley and Wilson, 1982
4	Hexamerins	*Spodoptera litura*	Involved in metamorphosis and reproduction	Tang et al., 2010
5	Lipophorin and lipid transfer particle (LTP)	*Manduca sexta, Locusta migratoria, Musca domestica, Periplaneta americana, Bombyx mori, and Rhodnius prolixus*	Involved in lipophorin function and transfer of lipids	Yokoyama et al., 2013
6	G proteins	*Helicoverpa armigera*	Involved in signaling	Hong-Hua et al., 2010
7	Heat shock proteins (hsp90, hsp70, and hsp60)	*Chilo suppressalis*	Involved in protein metabolism	Lu et al., 2014
8	Valine-rich midgut proteins	*Manduca sexta*	Involved in digestion of the plant diet	Odman-Naresh et al., 2013
9	Peptidoglycan recognition proteins (PGRPs)	*Culicidae (Armigeres subalbatus)*	Involved in recognition and binding of peptidoglycan	Wang et al., 2012
10	Inhibitor of apoptosis protein (IAP)	*Spodoptera frugiperda*	Involved in suppression of apoptosis	Huang et al., 2000
11	Cuticle proteins (MsCP29) (MsCP30/11)	*Manduca sexta*	Involved in molting	Csikos et al., 1999
12	Pupal cuticular proteins (CPs)	*Tenebrio molitor*	Involved in molting	Lemoine et al., 1993
13	Cytoskeletal proteins zeelin1 and zeelin2	*Lethocerus*	Involved in formation of cytoskeleton	Ferguson et al., 1994
14	Kettin and projectin	*Bombyx mori Danaus plexippus*	Involved in high resting stiffness of indirect flight muscles	Ayme-Southgate et al., 2013
15	Collagen	*Delia antiqua*	Fibrous protein, involved in diapause	You-Jin et al., 2014
16	Sericin	*Bombyx mori*	Involved in growth of corneal cells	Chirila et al., 2013
17	Fibrion	*Bombyx mori*	Fibrous protein, involved in development of eye cell	Chirila et al., 2013
18	Juvenile hormone binding protein (JHBP)	*Galleria mellonella*	Involved in transport of hormone	Zalewska et al., 2009
19	Chorion proteins	*Lepidopteran*	Involved in formation of eggshell	Giannopoulos et al., 2013
20	Vitelline membrane proteins (VMPs)	*Bombyx mori, Manduca sexta, Danaus plexippus, Heliconius melpomene*	Involved in membrane formation in egg shell	Xu et al., 2012
21	TGF-alpha-like protein	*Drosophila*	Involved in establishment of anterior-posterior and dorsal-ventral polarity through signal transduction pathway	Neuman-Silberberg and Schüpbach, 1996
22	Metallothioneins (MT)	*Orchesella cincta*	Involved in binding of heavy metals	Hensbergen et al., 2000
23	Transferrin (GmmTsf1) and Ferritin	*Glossinidae (tsetse fly)*	Involved in transport and metabolism of iron	Strickler-Dinglasan et al., 2006
24	Aquaporins	*Aedes aegypti Drosophila melanogaster Polypedilum vanderplanki Cicadella viridis*	Involved in transport of water molecules	Spring et al., 2009
25	Peritrophin	*Holotrichia oblita*	Involved in protection of the midgut epithelial cells from pathogens	Liu et al., 2014
26	Cecropins	*Musca domestica*	Involved in fat body and hemocytes	Xu et al., 2007
27	Glossina morsitans morsitans yolk protein 1 (GmmYP1)	*Glossinidae (tsetse fly)*	Major protein found in tsetse “milk” secretions	Attardo et al., 2006
28	Basic-helix-loop-helix-PAS protein (bHLH-PAS)	*Drosophila spineless*	Transcription factor involved in control of antennal and tarsal development	Emmons et al., 1999
29	SAS6 (spindle assembling abnormal protein 6)	*Delia antique*	Involved in chromosome separation, folate metabolism and other physiological processes	You-Jin et al., 2014

## Insecticides targeting key proteins/enzymes

After the discovery of insecticidal activity of DDT and lindane, the first generation synthetic insecticides, the chemical compounds targeting key insect proteins/enzymes revolutionized the insects control methods. Few insecticides like insect growth regulators (IGRs) (juvenile hormone analogs and chitin synthesis inhibitors) and miscellaneous active ingredients (borates, energy inhibitors, and dehydrating dusts) affects the water balance, oxygen metabolism, insect's molting or maturation process, and other aspects of physiology (Nauen and Bretschneider, 2002). Insecticides which act on insect's central nervous system are called neurotoxins. Various components of the acetylcholine system, GABA-gated chloride, and sodium channel modulators are targeted by neurotoxins. Organophosphates (OPs), neonicotinoids, and spinosyns inhibit the acetylcholinesterae (AchE) by removing the neurotransmitter acetylcholine (Ach) from its receptor in the post-synapse nerve and cause overstimulation of the nerve cell and finally insect death (Nauen and Denholm, 2005; Suiter and Scharf, 2011). Further, GABA-gated chloride channels located in the insect central nervous system and peripheral nerves are important target sites for insecticides. Chlorinated cyclodienes (e.g., Dieldrin, Endosulfan-Phasar, Thiodan) are class of insecticides which act on central nervous system. Fipronil binds and blocks the GABA receptor on the post-synapse nerve cell and prevents the influx of chloride ions and leads to the rapidand uncontrolled nerve firing throughout the insect nervous system (Nauen and Bretschneider, 2002). Pyrethroids and oxadiazines are insecticides which interact with sodium channels, the most important molecular target site in insect nervous system. These insecticides disrupt normal nerve function by regulating the sodium channels resulting tremors and quick death (Wing et al., 1998).

Many compounds known to interfere with insect development, in particular with ecdysis (skin shedding), chitin deposition, and entire process of molting are called IGRs (Hoffmann and Lorenz, 1998). IGRs normally interrupt critical physiological functions associated with normal insect growth, development, and reproduction. The IGRs like diflubenzuron, buprofezin, pyriproxyfen, hydroprene, methoprene, fenoxycarb, benzoylphenyl ureas, fenoxycarb, pyriproxyfen, and cryomazine are important insecticides being used for control of many insects (Beckage, 2000). Another class of IGRs is chitin synthesis inhibitors involved in hampering of biochemical pathway of chitin synthesis. Briefly, several chemical and bio insecticides have been developed targeting few of the proteins or enzymes of insects (Table 3). It is interesting to note that some of these targets have been used in RNAi mediated resistance development also. However, it is important to explore more effective targets in years to come which can be achieved by characterizing the role of insect proteins/enzymes in a genome-wide scale.

**Table 3:** **Mode of action, target site, and primary route of entry of commonly used insecticide classes**.

**Chemical class/group**	**Active ingredients/examples of trade names**	**Mode of action**	**Targeted site/process**
**INSECTICIDES THAT TARGET THE INSECT NERVOUS SYSTEM**			
Carbamates	Aldicarb (Temik), Bendiocarb (Garvox), Carbofuran (Furadan), Carbosulfan (Advantage), Promecarb (Carbamult), Methiocarb (Mesurol)	Acetyl cholinesterase inhibitor	Nerve synapse
Organo Phasphates	Acephate (Orthene), Chlorpyrifos (Lorsban), Disulfoton (Di-Syston), Fenthion (Baycid), Monocrotophos (Azodrin), Phorate (Thimet)	Acetyl cholinesterase inhibitor	Nerve synapse
Pyrethrins/Pyrethoids	Bifenthrin (Brigade), Cypermethrin (Ripcord), Fluvalinate (Mavrik), Permethrin (Talcord)	Sodium channel modulators	Axon of nerve
Neonicotinoids	Acetamiprid (pristine), Clothiamidin (poncho), Imidacloprid (Advantage), Thiacloprid (calypso)	Acetylcholine receptor agonist (mimic)	Nerve post-synapse
Avermectins	Abamectin B1 (Advert, Agri-Mek, Vertimec), Emamectin benzoate (Denim, Proclaim)	Chloride channel activators	Nerve post-synapse
Oxadiazins	Indoxacarb (Avaunt, Steward)	Voltage-dependent sodium channel blocker	Axon of nerve
Spinosys	Spinosad (Entrust, NaturaLyte, SpinTor, Success, Tracer)	Nicotinic acetylcholine receptor agonists (allosteric)	Nerve post-synapse
Phenylpyrazoles	Fipronil (Regent)	GABA-gated chloride channel antagonists	Nerve post-synapse
**INSECTICIDES THAT DO NOT TARGET THE INSECT NERVOUS SYSTEM**			
Juvenile hormone analogs	Fenoxycarb (Comply) Hydroprene (GenTrol) Methoprene (Apex) Pyriproxyfen (Archer, Knack)	Mimic juvenile hormone action	Growth and development
Chitin synthesis inhibitors	buprofezin (Applaud), cyromazine (Trigard)	Chitin synthesis inhibitor	Exoskeleton
Pyrroles	Chlorfenapyr (Alert, Pirate, Pylon)	Oxidative phosphorylation disruption—uncoupler	Metabolic processes/Energy production
Fumigant (sulfuryl fluoride)	Sulfuryl fluoride (Vikane)	Disruption of the glycolysis and fatty acid cycles	Metabolic processes/energy production
Borates	Borax(Boracide) Boric acid(Bonide Roach Powder, Enforcer Roach Ridd)	Miscellaneous non-specific (multi-site) inhibitors	Cells

*Table is derived from data developed and organized by the Insecticide Resistance Action Committee IRAC (2014), http://www.irac-online.org/modes-of-action/, and Brown and Ingianni, 2013*.

## RNA interference: powerful tool to target key genes of insects

RNA interference is an evolutionarily conserved, homology based gene regulation and defense mechanism found in most, if not all eukaryotic organisms. It operates through non-coding small RNA molecules, which recently gained wide spread attention, as molecular switches in complex gene regulatory networks. Several classes of small RNAs are reported which are processed through different enzymatic machinery (Agarwal and Mangrauthia, 2013). These small RNAs are central molecules of RNAi pathway and perform various functions in cells. Among the small RNAs, small interfering RNAs (siRNAs) are associated with defense against parasites, heterochromatin formation, transposons and transgenes silencing and post transcriptional gene silencing. The siRNAs are produced from double stranded RNA (dsRNA) with the help of RNaseIII enzyme dicer and its homologs. These siRNAs loaded with RISC help in identifying the target RNA through Watson-Crick homology. RISC, a multiprotein complex possess endonucleases such as Argonaute and its homologs to cleave the target RNA. This phenomenon has been utilized to attain resistance in plants against pests by producing dsRNAs homologous to the important pest genes. Numerous studies indicate that target gene silencing by RNAi could lead to either insect death or hampered growth and development. The first success of RNAi in insects was achieved with the model organism, the fruit fly *D. melanogaster* (Kennerdell and Carthew, 1998; Brown et al., 1999) whose genome sequence was published by Adams et al. (2000).

RNAi has been considered as a potential strategy for insect pest control (Zhang et al., 2013b). In past few years, it has become one of the most exciting discoveries of molecular biology, for its high specificity, accuracy, and hereditability. The technologies based on RNAi pathways have shown its potential in a very limited time and in wide range of field applications. The successful history of RNAi technology in crop protection against viruses inspired to achieve insect control by knock down of key enzymes or proteins of insects. The majority of studies on RNAi for insect control have been engrossed on the insect midgut as it is considered as most effective target for the gene silencing. The success of RNAi technology primarily relies on identification of suitable candidate genes to utilize them as targets. Several enzymes and proteins of insects have been identified and characterized (discussed above) which can be useful targets of RNAi. In plants, RNAi is often achieved by a transgene that produces hairpin RNA (hpRNA) with a dsRNA region (Waterhouse and Helliwell, 2003).

There are two primary conditions that need to be addressed to produce dsRNA expressing transgenic plants targeted against pest genes. First, it is important to ensure that adequate amount of dsRNA triggers is produced in plants and ultimately delivered to the pest body to produce an RNAi effect. Second, silencing of target gene through RNAi must cause insect mortality or any other phenotypic change such as preventing feeding, hampered development and/or reproduction. The first condition can be satisfied atleast in those insects which consume plant organs. Expression of dsRNA in plants can be optimized by selecting the specific promoters and other regulatory elements such as enhancers or suppressors. However, the second condition is not easy and purely depends on selection of target gene which determines the effect of RNAi on insect phenotype. Further, some studies have shown that transgenic plants expressing dsRNAs directed against insect genes showed enhanced resistance suggesting that RNAi was still active insect body. However, it is important to analyze the RNAi effects in case by case, as absence of RNAi machinery has been reported in few insects and other organisms. Further, in some cases, though RNAi machinery is present, but it does not show systemic effects (Gatehouse and Price, 2011).

RNAi has advantage over the existing technologies of insect control as it can target either specific insect or group of insects depending on the dsRNA trigger sequence. If RNAi has to be produced against specific insect, a unique and specific gene sequence should be selected for dsRNA production. In order to produce RNAi transgenic plants for multiple insect resistances, gene sequence conserved in those insects need to be identified for targeting through RNAi. Thus, selection of gene sequence for dsRNA production determines the spectrum of resistance in transgenic plants. Further, multiple insect resistance plants can also be obtained by pyramiding of gene constructs either at the time of transformation or through breeding approach.

**Figure 1:**
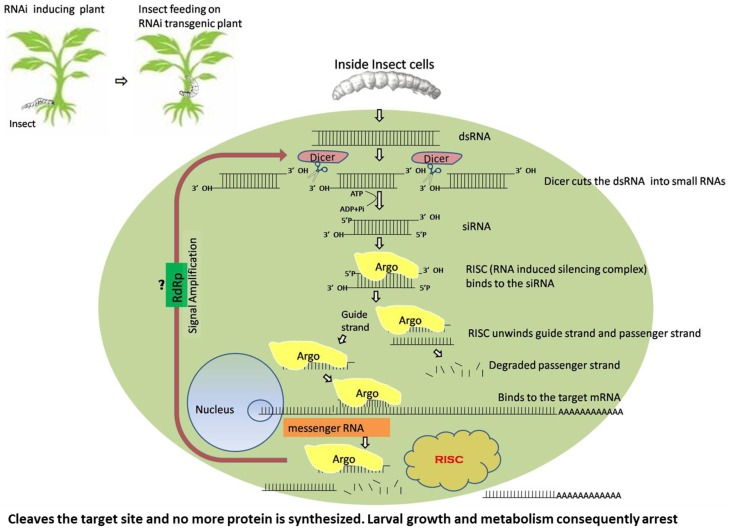
**Systematic illustration of gene silencing by RNAi inside the insect cells**. Double stranded RNA (dsRNA) of target genes are formed, which trigger RNAi machinery. dsRNAs are processed by RNAse III enzyme Dicer to synthesize 21 nucleotide small interfering RNAs (siRNAs). RNAi inducing silencing complex (RISC) binds these siRNAs. The guide strand of siRNAs helps RISC to target the corresponding mRNA. Argonaute protein (Argo) present in RISC complex cleaves the target mRNA. The cleaved target mRNA is amplified by RNA dependent RNA polymerase (RdRp) to form dsRNAs, which enter in RNAi pathway and amplify the signal. However, the signal amplification step in insects is not yet very well understood.

Though RNAi is a conserved phenomenon, it shows difference in mode of action in different insect orders. Three genes, *sid*-1, −2, and −3, were identified in *C.elegans* which are necessary for systemic RNAi effects in nematodes (Winston et al., 2002). The encoded protein of *sid-1* was characterized as a trans-membrane protein that functions as a pore or channel that transports dsRNA or siRNA into and out of cells. SID1 or its homolog has not been identified in *D. melanogaster* which does not show systemic RNAi effects. Further, systemic nature of RNAi is also determined by presence of absence of an enzyme RNA dependent RNA polymerase (RdRP) which helps in amplifying the silencing signal. The characteristic domains of RdRP have not been identified in insects genome, however, *Tribolium confusum*, a red flour beetle shows a strong, systemic, and trans-generational RNAi response. Interestingly, *Tribolium* shows RNA amplification through a mechanism possibly distinct from that observed in *C. elegans*. A genome-wide analysis suggested that several components of the RNAi are different between *C. elegans* and *T. confusum* (Tomoyasu et al., 2008). Initially, the SID1 was thought to be the determining factor of systemic nature of RNAi in insects given the fact that three homologs of *sid-1* were identified in *T. confusum* (showing systemic RNAi), while it was absent in Drosophila (non-systemic RNAi). Later, with the help of other insect genomes, it was proposed that the presence of *sid-1* homologs and systemic RNAi responses does not have direct correlation, suggesting that transport of dsRNA into and out of cells differ between insects and nematodes, and probably between the insect orders (Tomoyasu et al., 2008). Beside the *Tribolium*, other insects e.g., the wasp *Nasonia vitripennis* also have shown the transfer of dsRNA from one generation to another, suggesting the presence of systemic RNAi (Lynch and Desplan, 2006). Silencing of genes expression through RNAi has been demonstrated in several insect orders such as Diptera, Lepidoptera, Coleoptera, Orthopetra, Hymenoptera, Blattodea, and Hemiptera (Misquitta and Paterson, 1999; Bucher et al., 2002; Rajagopal et al., 2002; Amdam et al., 2003; Dong and Friedrich, 2005; Cruz et al., 2006; Mutti et al., 2006). However, the efficiency of the RNAi based genes suppression is variable in different insect species due to presence/absence of systemic RNAi and several other silencing components. Infact, transgenic plants producing dsRNAs directed against genes function in Lepidoptera, Coleoptera, and Hemiptera pests have shown encouraging results which need to be translated as commercial products against several other important crop insects (Baum et al., 2007; Gordon and Waterhouse, 2007; Mao et al., 2007; Chen et al., 2010; Agarwal et al., 2012). The biochemical machinery and steps involved in RNAi based insect resistance are described in Figure 1.

## Proven RNAi-based approaches of insect resistance

Hydroxy-3-methylglutaryl coenzyme A reductase (HMG-CoA reductase; HMGR) is a key enzyme in the mevalonate pathway of insects which is a potential RNAi target in case of *Helicoverpa armigera*. Silencing of HMGR using systemic RNAi inhibited the fecundity of the females, ovipostion, and significantly reduced vitellogenin (Vg) mRNA levels (Wang et al., 2013). RNA interference mediated down-regulation of cathepsin D of the silkworm *Bombyx mori* (*BmCatD)* resulted in the arrest of larval-pupal transformation (Gui et al., 2006). Knockdown of polygalacturonase (PG) gene in *L. lineolaris* bugs through injection with PG1 dsRNA reduced the expression of PG1 (Walker and Allen, 2010). RNAi based silencing of *aminopeptidase-N* (APN) and cadherin of *H. armigera* showed reduction of *HaAPN1* transcript levels after dsRNA incubation. This treatment also resulted in a corresponding decrease in the protein expression levels (Sivakumar et al., 2007). In another study, Rajam and coworkers silenced the acetylcholinesterase gene of *H. armigera* by feeding siRNAs which hampered larval growth (Kumar et al., 2009). Bautista et al., 2009 studied the influence of silencing the cytochrome P450 gene CYP6BG1 in permethrin resistance in diamondback moth (*Plutella xylostella*) through oral delivery of dsRNAs. In another report, *cytochrome P450 6B46 (CYP6B46)* -silenced larvae of *Manduca sexta* showed impaired mechanisms of passing ingested nicotine from the midgut to the hemolymph, thus making them susceptible to predators(Kumar et al., 2014). In yet another report, the cotton bollworm larvae were fed on RNAi transgenic leaves, showing reduced levels of cytochrome P450 (CYP6AE14) mRNA and retarded larval growth (Mao et al., 2007). NADPH-cytochrome P450 reductase (CPR) plays a central role in cytochrome P450 action of *Cimex lectularius*. The injection of dsRNA of *ClCPR* gene successfully suppressed the gene expression in all body parts indicating that the RNAi effect in bed bugs is systemic (Zhu et al., 2012). In a very short span of time, several efforts have been made to develop insect resistance using RNAi based targeting of key insect genes (Table 4).

**Table 4:** **List of genes encoding key enzymes/proteins used in RNAi mediated insect resistance**.

**S.No**	**Target gene**	**Type of RNAi construct**	**Crop/plant**	**Delivery method**	**Reported insects**	**References**
1.	Adenylatekinase 2 (AK2)	dsRNA	—	Feeding	*Helicoverpa, Drosophila*	Chen et al., 2012
2.	Vacuolar-ATPase A V-ATPase E	dsRNA	—	Ingestion and injection	*Peregrinus maidis, and Bemisia tabaci Diabrotica virgifera virgifera*	Baum et al., 2007; Upadhyay et al., 2011; Yao et al., 2013
3.	α-Tubulin	dsRNA	—	Feeding	*D.virgifera virgifera*	Baum et al., 2007
4.	Carboxylesterases (CarEs)	dsRNA	—	Injection	*Locusta migratoria*	Zhang et al., 2014
5.	Carboxylesterase gene *EposCXE1* and pheromone binding protein gene *EposPBP1*	dsRNA	—	Oral delivery /feeding	*Epiphyas Postvittana*	Turner et al., 2006
6.	Catalase (CAT)	dsRNA	—	Injection	*Spodoptera litura*	Zhao et al., 2013
7.	Superoxide dismutase	dsRNA	—	Injection	*Culex pipiens*	Sim and Denlinger, 2011
8.	Laccase 2 (Lac2)	dsRNA	—	Injection	*D.virgifera virgifera*	Alves et al., 2010
9.	Arginine kinase	dsRNA	—	Feeding	*Phyllotreta striolata*	Zhao et al., 2008
10.	Allatotropin	dsRNA	—	Injection	*Tribolium castaneum*	Abdel-latief and Hoffmann, 2014
11.	Allatostatin C	dsRNA	—	Injection	*T. castaneum*	Abdel-latief and Hoffmann, 2014; Lungchukiet et al., 2008
12.	Trehalose phosphate synthase (TPS)	dsRNA	—	Feeding	*Nilaparvata Lugens*	Chen et al., 2010
13.	Vitellogenin protein	dsRNA	—	Injection	*S.litura*	Shu et al., 2011
14.	Cytochrome P 450 CYP6BG1	dsRNA	—	Feeding	*Plutella xylostella*	Bautista et al., 2009
15	CYP6B6	dsRNA	—	Feeding	*H. armigera*	Zhang et al., 2013a
16	CYP6AE14	pBI121- pCAMBIA1300	Arabidopsis and Tobacco	Feeding	*H.armigera*	Mao et al., 2007
17	Aminopeptidase N	dsRNA	—	Injection	*S.litura*	Rajagopal et al., 2002
18	3-hydroxy-3-methylglutaryl coenzyme A reductase (HMG-CoA reductase; HMGR) gene	dsRNA	—	Injection	*H. armigera*	Wang et al., 2013
19	Tryptophan oxygenase	dsRNA	—	Injection	*Plodia interpunctella*	Lorenzen et al., 2002
20	HaHR3	pCAMBIA2300-35s-OCS	Tobacco	Feeding bioassays and transgenic expressing hairpin RNAs	*H.armigera*	Xiong et al., 2013
21	Hexose transporter gene *NlHT1*	pCU	Rice	Hairpin RNAi construct.	*N.lugens*	Zha et al., 2011
22	Carboxypeptidase gene *Nlcar*	pCU	Rice	Hairpin RNAi construct.	*N.lugens*	Zha et al., 2011
23	Circadian clock gene *per*	dsRNA	—	Injection	*Gryllus Bimaculatus*	Moriyama et al., 2008
24	Salivary nitrophorin 2 gene *NP2*	dsRNA	—	Injection	*Rhodnius prolixus*	Araujo et al., 2006
25	Eye color gene vermilion	dsRNA	—	Injection	*Schistocerca Americana*	Dong and Friedrich, 2005
26	Trypsin-like serine protease gene *Nltry*	Hairpin RNAi construct	Rice	.	*N.lugens*	Zha et al., 2011
27	Circadian clock gene	dsRNA	—	Injection	*S. littoralis*	Kotwica et al., 2009
28	β-actin gene	dsRNA	—	Injection	*S. littoralis*	Gvakharia et al., 2003
29	Glutathione-S-transferase gene GST1	dsRNA	—	Feeding	*H. armigera*	Mao et al., 2007
30	Chitin synthase gene	dsRNA	—	Injection	*S. exigua*	Chen et al., 2008
31	Chitin synthase genes TcCHS1 and TcCHS2	dsRNA	—	Injection	*T. castaneum*	Arakane et al., 2005
32	Chitinase like proteins TcCHT5, TcCHT10, TcCHT7, and TcIDGF4	dsRNA	—	Injection	*T. castaneum*	Zhu et al., 2008
33	Polygalacturonase	dsRNA	—	Injection	*Lygus lineolaris*	Walker and Allen, 2010
34	Acetylcholinesterase	dsRNA	—	Feeding	*H. armigera*	Kumar et al., 2009

## Challenges of RNAi based insect resistance

Several fine tuning steps need to be taken up before the success of insect control through RNAi can be fully realized. More intensive research is needed to identify improved siRNA delivery methods, prediction of effective target genes and siRNAs, identifying the conserved gene sequences, stability and durability of gene silencing effects. Further, important factors determining the success of RNAi in insect control would be identifying the RNAi machinery in important insect species, identifying novel and effective gene targets of RNAi, identifying the correct life stage of insect to be targeted, identifying the right tissue of plants for siRNA biogenesis and optimization of required concentration of siRNAs to kill insect, nullifying the off target effects of siRNAs in useful and non-targeted organisms, and biosafety assays of RNAi products.

More gene orthologs involved in key metabolic pathways of insects should be identified and their roles need to be characterized. Use of advanced genomics and proteomics tools will hasten the process of identification of crucial genes/ proteins. Generally, the candidate targets of RNAi may be any gene showing detrimental lethal phenotype on insect life cycle. The genome sequence information of insects has currently around 340,000 of total sequences for beetles (http://www.ncbi.nlm.nih.gov/nuccore/). Only eight coleopteran species have more than 5000 sequences deposited- *Pogonus chalceus* (65779), *Dendroctonus ponderosae* (41429), *Rhynchophorus ferrugineus* (27014), *Dendroctonus frontalis* (20987), *T. castaneum* (16808), *Ips typographus* (14810), *Agrilus planipennis* (12018), and *A. grandis* (5705), (Firmino et al., 2013; i5K Consortium, 2013). More sequences from diverse insect species will help in identifying useful gene targets of RNAi. Development of insect cell lines especially from midgut microvillar epithelial cells will facilitate in deciphering the dsRNA uptake mechanism. It will help in developing effective siRNA delivery methods. Development of non-reactive material for coating of siRNAs will further enhance the scope of delivery. Target siRNAs can be delivered through nano particles coated with dsRNA to enhance its uptake by the gut and in turn the efficiency of the silencing. To make it more stable and heritable insect control tool, siRNA producing gene constructs can be developed which can become resources to produce novel insect resistant transgenic crops. Here, siRNA production can be optimized by using either tissue or stage specific promoters, and enhancers. Despite significant progress in understanding the siRNA uptake mechanism, there is a need to develop effective artificial diets for some important insects like yellow stem borer of rice. It will augment the testing and validation of the RNAi targets. Further, tagging the siRNA with florescent dyes will help to track the siRNAs movement in the insect body.

Although RNAi is conserved pathway among several eukaryotes but it has diversified a lot in the course of evolution (Shabalina and Koonin, 2008). Infact, in some organisms such as *Saccharomyce cerevisiae*, and *Ustilago maydis*, the entire RNA silencing machinery appears to be lost (Nakayashiki and Nguyen, 2008). Therefore, it is necessary to probe more into the elements of RNAi machinery in different insects species before applying RNAi in a given insect.

### Delivery methods of RNAi

RNAi shows its impact once the dsRNA is delivered into the cell (Yu et al., 2013). The dsRNA delivery methods used till today are oral feeding, microinjection, RNAi transgenic crops, soaking and transfection methods (Huvenne and Smagghe, 2010). The oral delivery of dsRNA in insects is one of the efficient approaches of RNAi. It was first demonstrated in *C. elegans*, (Timmons and Fire, 1998). Recently, it was shown in *H.armigera* with siRNAs designed from AChE (Kumar et al., 2009). Though it is simple method of dsRNA delivery, it has many disadvantages like heritability and stability issues, and many of the insects do not have the diet to complete its life cycle. Microinjection is another most commonly used method for dsRNA delivery in insects (Arakane et al., 2005; Suzuki et al., 2008). Though it is a precise way of dsRNA delivery, it requires technical expertise and specific equipments, so it is not much popular in many insect systems.

In recent years, soaking the organism into a dsRNA solution seems to be a popular method for triggering RNAi response mainly because of its convenient operation (Yu et al., 2013). The first experiment regarding soaking was reported by Tabara group in which they found that specific RNAi was induced by simply soaking of *C. elegans* in the dsRNA solution (Tabara et al., 1998). Thus, this technique was applied for large-scale analysis of gene function in this species to accomplish high-throughput RNAi (Maeda et al., 2001). The dsRNA delivery methods such as oral feeding, microinjection, soaking, and transfection are important in studying RNAi in insects, however, these methods do not seem to be of great help as concerns the defense of plants against insects.

RNAi trigger can also be delivered in insects through transgenic plants. In a recent study, pCAMBIA-RNAi-*HaHR3* construct was developed and transformed into tobacco (*Nicotiana tabacum*) expressing the *HaHR3* mRNA hairpin which resulted developmental deformity and larval lethality of *H. armigera* (Xiong et al., 2013). Tobacco and Arabidopsis transgenics expressing CYP6AE14 -siRNAs of the *H. armigera* inhibited expression of the CYP6AE14 mRNA in this lepidopteron pest. The suppression of CYP6AE14 expression in these insects caused increased sensitivity to the natural defense compound, gossypol, produced by the cotton plant (Mao et al., 2007). In another study, transgenic corn plants expressing V-ATPase-dsRNA of the western corn rootworm showed resistance to feeding damage by this lepidopteron pest (Baum et al., 2007). Transgenic rice developed based on RNAi of three separate genes expressed in the midgut of the brown plant hopper, *Nilaparvata lugens* Stål showed a reduced expression of target genes in this insect (Zha et al., 2011). Further, transgenic Arabidopsis targeting receptor of activated kinase (Rack-1) gene was developed to induce RNAi in the gut of the aphid *Myzus persicae* (Pitino et al., 2011). In yet another study, transgenic tobacco expressing dsRNA of v-ATPaseA gene showed enhanced resistance against whitefly (Thakur et al., 2014). The persistence and trans-generational effects of plant-mediated RNAi based on Rack1, MpC002, and MpPIntO2 genes was reported in the green peach aphid (GPA) *M. persicae* using transgenic Arabidopsis (Coleman et al., 2015). Overall, delivery of dsRNAs through transgenic approach has been successful in some cases while it was discouraging in other reports. Of course, it has advantage over other methods of delivery in terms of stability, heritability and optimization of dsRNA expression in specific tissues and quantity. However, this method of dsRNA delivery relies on feeding behavior of insects on transgenic crops. Therefore, it is necessary to examine and identify appropriate dsRNA or siRNA delivery methods in a case wise manner.

### Off-targeting

Although, initially gene silencing by RNAi was believed to be specific, recent reports have shown that off-targeting can widely occur during RNAi (Xu et al., 2006). It was reported in Triatomid bug (*R. prolixus*) while silencing nitroporin 2 (Araujo et al., 2006). Though, siRNA sequences are highly specific to the target genes, non-specific hybridization of siRNAs with non-target transcripts can induce unintended effects (Jackson et al., 2003). Therefore, it is important to ensure that the dsRNA and corresponding siRNA sequences do not exert off-target effects that negatively impact host plant physiology, potential non target host colonizers and/or mammals that feed on the modified crop (Koch and Kogel, 2014).

Sequence similarity between siRNA and target gene, mRNA sequence selected for RNAi, size of siRNA, and transitive RNAi determine off-target silencing. Use of highly specific sequences in siRNA and controlling their expression through specific and inducible promoters can minimize off targeting effects. Further, off targeting effects can be minimized using artificial microRNA technology that provides a way to design specific siRNAs instead of using additional sequences (Schwab et al., 2006; Warthmann et al., 2008; Ai et al., 2011). While selecting the targets, it is important to select the genes which are unique and do not occur as gene family or homologous genes. Also, silencing of genes associated with regulatory functions and multiple metabolic pathways such as transcription factors or signaling molecules may lead to unintended effects on host. Therefore, it is important to examine the off target effects of RNAi at multiple levels. First, designing of siRNA to silence a particular gene in any organism must ensure that it does not target any other mRNA of its own. Second, off targeting of siRNA need to be checked in other closely associated plants/crops which can be cross pollinated. Third, possible off targeting impacts need to be analyzed on microorganisms and insects cohabiting with RNAi plants. Particularly, it needs to be assessed for useful and non-targeted organisms. In order to make sure that other non-harmful insects are not killed, selection of target genes through screening would be appropriate approach. Genes playing essential functions and unique to target insects need to be selected. House-keeping genes including those encoding various ribosomal proteins, and components of conserved systems such as proteasomes and transport or genes belonging to conserved multi-gene family may affect non-harmful insects. The insect genes whose products are necessary for detoxification of a secondary metabolite in the plant host may be appropriate. Here, developing a genome wide sequence and transcriptome databases of non-harmful insects for different ecologies will facilitate in selection of genes which will not affect non-target organisms. Fourth, off targeting also need be analyzed on animals grazing on such plants/crops. Most importantly, if RNAi is introduced in food crops, off targeting of siRNAs need to be checked against human genome. In order to achieve this, genome sequence data, transcriptome data, or EST databases of plants, animals, and microorganisms will be extremely important to search homologous off target sequences. However, the gene/genome sequences of most of the predator parasites are not available. Though, appreciable increase in genome and transcriptome databases has been witnessed after the commencement of next generation sequencing tools and advanced bioinformatics pipelines. Currently, the prediction of off targeting is done through bioinformatics approaches using the existing gene sequence databases (Agarwal and Mangrauthia, 2013). Therefore, it will be important to develop databases of different organisms which can be utilized to predict the possible off target effects. One such database has been developed called siRNA Scan (http://bioinfo2.noble.org/RNAiScan.htm) to identify potential off-targets in plants (Senthil-Kumar and Mysore, 2011).

## Future prospects

Tremendous progress has been achieved in using RNAi technology to understand gene regulation and function in humans and other animal systems. RNA interference has become an indispensable tool for loss-of-function studies across eukaryotes (Fellmann and Lowe, 2014). Applications of RNAi are extended to plant system for functional genomics. Advancement of this technology is also evident in developing resistance against viruses and fungi in some crop plants (Wani et al., 2010; Duan et al., 2012). However, use of this technology for insect control is in beginning and the progress is encouraging. Undoubtedly, it has several merits over the existing methods of insect control. To make this technology more viable and effective, intensive studies are required to understand the insect biology. High throughput genomics and proteomics tools need to be utilized effectively for detecting real candidate genes encoding key proteins or enzymes which are still not characterized in systems approach. Classical studies are required to develop the artificial diets and more effective siRNA delivery methods. Funding is required to generate sequence databases of beneficial and harmless insects and insect predators of important crop ecologies. International consortia to develop such sequence database can be formed. Though suppressors of RNAi pathway have been well documented in the virus system (Praveen and Mangrauthia, 2006; Mangrauthia et al., 2010), the research efforts are needed to see if such suppressors are existing in the insects also to counter the RNAi based plant defense. Though RNAi technology seems to be safer than protein based transgenic technology, still more studies on biosafety issues are required to popularize the technology which will help in bringing safer products into the environment and commercial markets. Importantly, RNAi needs to be designed to limit inter-species activity by selecting the appropriate genes and dsRNA trigger sequences. Here, genome, transcriptome and EST databases will play crucial role in identifying specific targets of RNAi. In particular, the dsRNA trigger sequences need to be cross checked with human genome and transcriptome considering that many of the putative insect targets may also be important in human (patho) physiology.

### Conflict of interest statement

The authors declare that the research was conducted in the absence of any commercial or financial relationships that could be construed as a potential conflict of interest.
